# Autonomous Nervous Response During Sedation in Colonoscopy and the Relationship With Clinician Satisfaction

**DOI:** 10.3389/fmed.2021.643158

**Published:** 2021-06-16

**Authors:** Alexander Hann, Sascha Gruss, Sebastian Goetze, Niklas Mehlhase, Stephan Frisch, Benjamin Walter, Steffen Walter

**Affiliations:** ^1^Department of Internal Medicine II, Gastroenterology, University Hospital Wurzburg, Wurzburg, Germany; ^2^Department of Internal Medicine I, University Hospital Ulm, Ulm, Germany; ^3^Department of Psychosomatic Medicine and Psychotherapy, University Hospital of Ulm, Ulm, Germany

**Keywords:** colonoscopy, biosignals, autonomic nervous system, propofol, sedation

## Abstract

**Background:** Nurse assisted propofol sedation (NAPS) is a common method used for colonoscopies. It is safe and widely accepted by patients. Little is known, however, about the satisfaction of clinicians performing colonoscopies with NAPS and the factors that negatively influence this perception such as observer-reported pain events. In this study, we aimed to correlate observer-reported pain events with the clinicians' satisfaction with the procedure. Additionally, we aimed to identify patient biosignals from the autonomic nervous system (B-ANS) during an endoscopy that correlate with those pain events.

**Methods:** Consecutive patients scheduled for a colonoscopy with NAPS were prospectively recruited. During the procedure, observer-reported pain events, which included movements and paralinguistic sounds, were simultaneously recorded with different B-ANS (facial electromyogram (EMG), skin conductance level, body temperature and electrocardiogram). After the procedure, the examiners filled out the Clinician Satisfaction with Sedation Instrument (CSSI). The primary endpoint was the correlation between CSSI and observer-reported pain events. The second primary endpoint was the identification of B-ANS that make it possible to predict those events. Secondary endpoints included the correlation between CSSI and sedation depth, the frequency and dose of sedative use, polyps resected, resection time, the duration of the procedure, the time it took to reach the coecum and the experience of the nurse performing the NAPS. ClinicalTrials.gov: NCT03860779.

**Results:** 112 patients with 98 (88.5%) available B-ANS recordings were prospectively recruited. There was a significant correlation between an increased number of observer-reported pain events during an endoscopy with NAPS and a lower CSSI (*r* = −0.318, *p* = 0.001). Additionally, the EMG-signal from facial muscles correlated best with the event time points, and the signal significantly exceeded the baseline 30 s prior to the occurrence of paralinguistic sounds. The secondary endpoints showed that the propofol dose relative to the procedure time, the cecal intubation time, the time spent on polyp removal and the individual nurse performing the NAPS significantly correlated with CSSI.

**Conclusion:** This study shows that movements and paralinguistic sounds during an endoscopy negatively correlate with the satisfaction of the examiner measured with the CSSI. Additionally, an EMG of the facial muscles makes it possible to identify such events and potentially predict their occurrence.

## Introduction

Colorectal cancer (CRC) is the third most common cause of cancer-related deaths in both men and women in the United States (1). In Germany, it even takes second place in the women's cancer statistics (2). A colonoscopy that can diagnose and treat precancerous lesions in the same session (3) is the recommended form of screening. Most of the colonoscopies performed worldwide use intravenous sedation (4–9). In Germany, the percentage of colonoscopies performed with sedation has increased in the past years by 91%. Regarding the drugs used for sedation, propofol has become the most common agent and was used in 97% of the procedures (10). Non-anesthesiologist propofol sedation, in particular nurse-administered propofol sedation (NAPS), is a safe and widely accepted procedure (11–13).

An adequate level of sedation during the performance of a colonoscopy is desirable both for routine cancer screening as well as interventional procedures. Accordingly, optimal anesthesia can improve quality measurements such as the polyp-detection rate, the cecal and the ileum intubation rate (14).

Another issue that can be addressed with an optimal sedation is the pain experienced during the endoscopic procedure, which is reported by up to almost a quarter of the patients. Especially propofol sedation presented superior efficacy compared to benzodiazepine-opioid sedation in the prevention of pain during colonoscopies (15).

Different efforts were made to improve the sedation procedure including computer-assisted propofol sedation (CAPS), which did not become the standard, however. One of the main issues that prevented the CAPS from being generally accepted might have been the high costs of establishing this method (16).

Even though sedation provides a high level of patient satisfaction and a low risk of serious adverse events, little is known about the examiner's satisfaction with this type of sedation (17). Thus, in this study we aimed to identify and measure, with the Clinician Satisfaction with Sedation Instrument (CSSI) (18), distinct and observer-reported events during the colposcopy, including movements and paralinguistic sounds such as moaning, that negatively influence the satisfaction of the examiner. In order to objectively detect such events, we additionally evaluated biosignals from the autonomic nervous system (B-ANS). It is known from biofeedback (19), affective computing (20) and automated pain recognition (21–24) that physiological signals can be used to operationalize sympathetic/parasympathetic regulatory mechanisms over time. B-ANS in this context are an electromyography of the corrugator and zygomaticus, skin conductance, the heart rate and the skin temperature. Thus, the secondary aim of this study was to identify physiological signals that make it possible to objectively detect pain events and further analyze which of those signals are capable of predicting such an event.

## Methods

### Patients

Consecutive patients scheduled for a colonoscopy were prospectively enrolled in the study after completion of a written, informed consent. Other inclusion criteria were age above 17 years, American Society of Anesthesiologists (ASA) physical status classification system I or II and procedure performed under propofol sedation. Exclusion criterion was pregnancy.

The study was approved by the local ethics committee (No 278/18) and registered on ClinicalTrials.gov under the identifier NCT03860779 prior to recruitment. A subgroup of the recruited patients served as a control group in a subsequent study on the influence of music for relaxation during colonoscopy (NCT04258800) (25).

### Measured B-ANS Parameters

**Figure 1:**
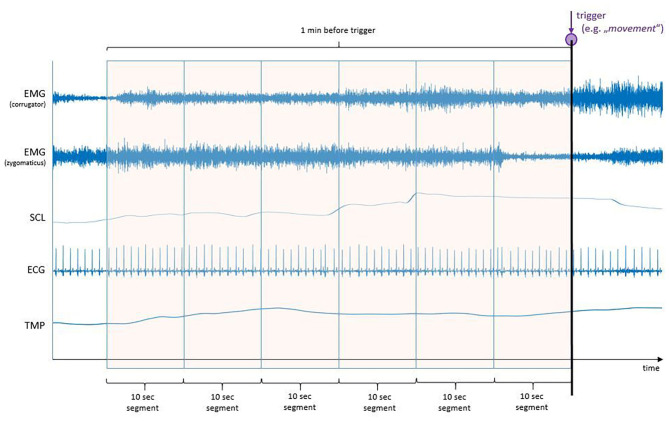
Biosignals of autonomous nervous systems (B-ANS) response during sedation in colonoscopy.

Prior to the start of the procedure, different electrodes were placed in order to record the various B-ANS (shown in Figure 1). The signals were recorded at a rate of 2,048 Hz by utilizing a Nexus amplifier and the corresponding BioTrace-Software (Mind Media, Herten, Netherlands):

1. Facial Electromyography (fEMG): Bipolar pairs of Ag/AgCl electrodes were utilized for measuring the fEMG activity. The electrodes were placed over the right corrugator supercilii and right zygomaticus major muscles.
2. Electrocardiography (ECG): Three single Ag/AgCl electrodes were utilized to measure the average cardiac action potential on the skin. One electrode was placed on the chest, ~6 cm below the right collarbone. The second electrode was placed on the left lower rib cage. The third electrode served as reference and was attached to the right-side waist next to the pelvic bone. It also served as reference for the fEMG.
3. Skin Conductance Level (SCL): Two electrodes were placed on the edge of the left hand to measure electrodermal activity (sweating).
4. Temperature (TMP): The temperature sensor was attached to the tip of the left little finger with medical tape.

### Procedure

The colonoscopy procedures were performed under NAPS using an Olympus colonoscope (CF-HQ190I, Olympus, Tokyo, Japan) with CO_2_ insufflation. Single propofol boluses were administered in order to reach the desired sedation level. Following the German and ESGE guidelines for sedation in gastrointestinal endoscopy, a moderate sedation level was targeted for colonoscopy procedures (26, 27). The additional administration of midazolam was allowed in accordance with national sedation guidelines (4).

### Observer-Reported Events

During the endoscopic procedure, various events were recorded on a tablet PC device (shown in Supplementary Figure 1) using a custom-made application. A single investigator recorded the events during the entire study period in order to avoid interobserver variation. Events included the start and end time point of the procedure and the time point of reaching the coecum or terminal ileum. Additionally, the modified Observer Assessment of Alertness and Sedation (MOAAS) was recorded every 3 min. The observer-reported pain events, which included movements and paralinguistic sounds, with a subdivided severity scale ranging from a value of one to three were recorded at corresponding time points.

### Questionnaires

All examiners were asked to fill out the Clinician Satisfaction with Sedation Instrument (CSSI) directly after the procedure (18). Additionally, patients filled out the Patients Satisfaction with Sedation Instrument (PSSI) at the end of an outpatient procedure, or in the ward in the case of an inpatient treatment (18). In both scores, a value of zero indicated very dissatisfied and 100 very satisfied.

### Signal Processing and Feature Extraction

**Figure 2:**
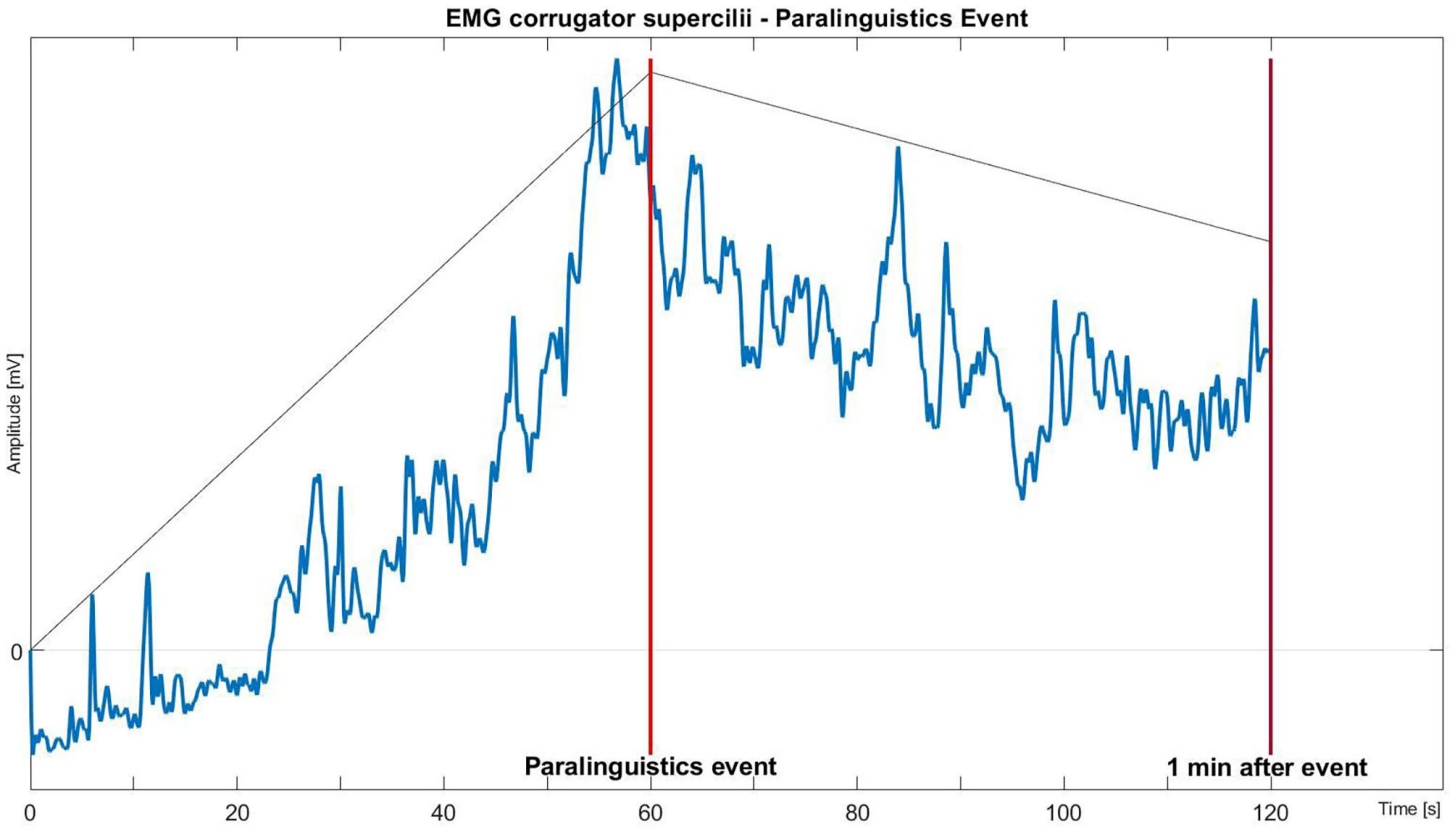
Biosignals of autonomous nervous signals (B-ANS) before one-minute windows (T1: 0–10 s, T2: 10–20 s, T3: 20–30 s, T4: 30–40 s, T5: 40–50 s, T6: 50–60 s) before relevant triggers (“*movement*” or “*paralinguistics*”) were cut out of the signals.

All signals were down sampled from 2,048 to 512 Hz to speed up processing. Afterwards, a Butterworth band-pass filter was applied to filter the raw fEMG (20–250 Hz) and the SCL (0.2–4 Hz) signals. The ECG and temperature signals were processed with moving average windows (for ECG: *n* = 67; for TMP: *n* = 513). Additionally, the fEMG and SCL signals were standardized in a person-specific manner. To obtain features for the statistical analysis, 1-min windows right before the relevant markers (“movement”, “paralinguistics”) were extracted from the signals and visually inspected for outliers and corrected, if necessary (shown in Figure 2).

Finally, features for all signals were extracted from six successive 10-s segments of each window.

For each segment, the following features were derived: ECG: A QRS-detection algorithm by Hamilton and Tompkins (28) was applied to find R-peaks in the segments. Subsequently, the average length of the R-to-R intervals was calculated (“meanRR” in ms).

SCL: The maximum value was determined (“maxSCL” in μS).

TMP: The mean was calculated (“meanTMP” in °C).

fEMG: The fEMG signals of each window were Hilbert-transformed (29), and absolute values were computed. In addition, the signals were further processed with a Butterworth low-pass filter with a cut-off frequency of 2 Hz. Lastly, the maximum value was determined for both the signal from the corrugator supercilii and the zygomaticus major (“maxCOR” and “maxZYG” in μV).

### Primary and Secondary Endpoints

The two primary endpoints included the correlation between the CSSI and the combined events of movements and paralinguistic sounds. The other primary endpoint included the prediction of observer-reported pain events, such as movements and paralinguistic sounds, using the B-ANS. The secondary endpoints were predefined as follows: The correlation between the CSSI and one of the following: sedation depth using MOAAS; frequency of sedation use; cecal intubation time; years of experience of the assisting nurse; polyps resected; total polyp resection time. Other secondary endpoints set after the start of the study were the correlation between the CSSI and one of the following: total propofol dose and amount relative to the duration of the procedure; total duration of the procedure; individual assisting nurse.

### Statistical Analysis

Statistical tests were performed using SPSS Statistics 25 (IBM, USA). The Spearman two-tailed test was used for bivariate non-parametric correlations. A *p*-value of < 0.05 indicated statistical significance. With regard to the repetition of measurements in the form of 6 points (T1: 0–10 s, T2: 10–20 s, T3: 20–30 s, T4: 30–40 s, T5: 40–50 s, T6: 50–60 s) in time prior to the observation (“*movement*”, “*paralinguistics*”)for all B-ANS, a Friedman test (with *ad-hoc* tests) for dependent samples was chosen. The relationship between B-ANS (60 s averaged prior to the observation) and the CSSI was examined with a non-parametric Spearman correlation.

## Results

### Patient Characteristics

From March 4, 2019 to July 1, 2019, 112 patients were prospectively included in the study (shown in Supplementary Figure 2) over a period of 29 days. The baseline characteristics showed an equal correlation between male and female participants (Table 1). The mean age was 54.1 years with a range from 18 to 89 years. The most common indications for the endoscopy were colorectal cancer screening and polyp removal (29%), followed by inflammatory bowel disease (27%) (Supplementary Table 1). All CSSI and 80% of the PSSI forms were completed. B-ANS were available for 98 (87.5%) participants.

**Table 1:** Baseline characteristics.

**Characteristic**	
Number of pts, *n*	112
Male, *n*(%)	57 (51)
Female, *n*(%)	55 (49)
Age, mean ± SD	54.1 ± 17.5
Completed CSSI, *n*(%)	112 (100)
Completed PSSI, *n*(%)	89 (80)

### Correlation Between CSSI and Observer Reported Movements and Paralinguistic Sounds

The primary endpoint regarding the correlation of the CSSI with observer-reported pain events, which included body movements and paralinguistic sounds, was significant (*r* = −0.318, *p* = 0.001). Thus and as expected, more movements and paralinguistic sounds during the endoscopic procedure result in a lower satisfaction with the sedation. The negative correlation was significant regarding movement events alone (*r* = −0,284, *p* = 0.002). The correlation between the CSSI and paralinguistic sounds alone showed just a trend toward significance (*r* = −0.179, *p* = 0.059). Interestingly, the patient satisfaction measured with the PSSI and observer-reported movements and paralinguistic sounds showed no significant correlation (*r* = −0.124, *p* = 0.248). Additionally, there was no correlation between the PSSI and the CSSI.

### Prediction of Observer Reported Movements and Paralinguistic Sounds by B-ANS

The second primary endpoint was to identify B-ANS that make it possible to detect and predict observer-reported pain events. With regard to the occurrence of the observed event, an evaluation was carried out for *movement* (*n* = 94) and *paralinguistic* sounds (*n* = 59). The further difference to the total number of available B-ANS of 98 resulted from the quality of the signals. Tables 2, 3 show the sympathetic autonomous nervous system (ANS) activity 60 s before the observation times with 6 time windows each (T1: 0–10 s, T2: 10–20 s, T3: 20–30 s, T4: 30–40 s, T5: 40–50 s, T6: 50–60 s). The following general effects were ascertained before the observation:

- Movement: fEMG_C: *X*^2^ = *85.32, p* ≤ *0.001, n* = *93*
- Movement: fEMG_Z: *X*^2^ = *72.50, p* ≤ *0.001, n* = *92*
- Paralinguistic: fEMG_C: *X*^2^ = *59.85, p* ≤ *0.001, n* = *59*
- Paralinguistic: fEMG_Z: *X*^2^ = *49.35, p* ≤ *0.001, n* = *58*

**Table 2:** Autonomous sympathetic activity before movement observation (*n* = 94) to six events T1-5 vs. T6.

	**fEMG_C (*****N*** **=** **93)**			**fEMG_Z (*****N*** **=** **92)**			**SCL (*****N*** **=** **82)**			**TMP (*****N*** **=** **94)**			**ECG (*****N*** **=** **85)**		
**Before observation**	**Mean**	**Wilcoxon**	***p*-value**	**Mean**	**Wilcoxon**	***p*-value**	**Mean**	**Wilcoxon**	***p*-value**	**Mean**	**Wilcoxon**	***p*-value**	**Mean**	**Wilcoxon**	***p*-value**
T1 (00–10 s)	0.678	T6–T1	0.000	0.431	T6–T1	0.000	−0.103	T6–T1	0.175	28.735	T6–T1	0.290	428.507	T6–T1	0.631
T2 (10–20 s)	0.524	T6–T2	0.000	0.361	T6–T2	0.000	−0.099	T6–T2	0.083	28.746	T6–T2	0.285	430.639	T6–T2	0.378
T3 (20–30 s)	0.651	T6–T3	0.000	0.453	T6–T3	0.000	−0.102	T6–T3	0.040	28.759	T6–T3	0.190	432.292	T6–T3	0.115
T4 (30–40 s)	0.653	T6–T4	0.000	0.445	T6–T4	0.000	−0.104	T6–T4	0.021	28.779	T6–T4	0.101	433.808	T6–T4	0.031
T5 (40–50 s)	0.843	T6–T5	0.000	0.541	T6–T5	0.000	−0.098	T6–T5	0.039	28.811	T6–T5	0.473	433.584	T6–T5	0.002
T6 (50–60 s)	1.421	–	–	0.724	–	–	−0.050	–	–	28.819	–	–	428.678	–	–

*fEMG_C, Electromyography-Corrugator; fEMG_Z, Electromyography-Zygomaticus; SCL, Skin Conductance Level; TMP, Temperature; ECG, Electrocardiogram*.

**Table 3:** Autonomous sympathetic activity before paralinguistic observation to six events T1-5 vs. T6.

	**fEMG_C (N** **=** **59)**			**fEMG_Z (N** **=** **58)**			**SCL (N** **=** **51)**			**TMP (N** **=** **59)**			**ECG (N** **=** **55)**		
**Before observation**	**Mean**	**Wilcoxon**	***p*-value**	**Mean**	**Wilcoxon**	**Mean**	**Wilcoxon**	***p*-value**	**Mean**	**Wilcoxon**	**Mean**	**Wilcoxon**	***p*-value**	**Mean**	**Wilcoxon**
T1 (00–10 s)	0.8143	T6–T1	0.000	0.5131	T6–T1	0.000	−0.0608	T6–T1	0.085	27.9784	T6–T1	0.757	439.5736	T6–T1	0.932
T2 (10–20 s)	0.8448	T6–T2	0.000	0.5522	T6–T2	0.000	−0.0199	T6–T2	0.245	27.9903	T6–T2	0.717	445.1325	T6–T2	0.960
T3 (20–30 s)	1.0500	T6–T3	0.000	0.5846	T6–T3	0.000	−0.0219	T6–T3	0.160	28.0106	T6–T3	0.582	444.6785	T6–T3	0.766
T4 (30–40 s)	1.1222	T6–T4	0.000	0.6515	T6–T4	0.013	−0.0259	T6–T4	0.057	28.0348	T6–T4	0.541	445.9821	T6–T4	0.726
T5 (40–50 s)	1.3029	T6–T5	0.000	0.6419	T6–T5	0.011	−0.0106	T6–T5	0.029	28.0531	T6–T5	0.910	447.7451	T6–T5	0.084
T6 (50–60 s)	1.7350	–	–	0.8775	–	–	0.0576	–	–	28.0807	–	–	448.0119	–	–

*fEMG_C, Electromyography-Corrugator; fEMG_Z, Electromyography-Zygomaticus; SCL, Skin Conductance Level; TMP, Temperature; ECG, Electrocardiogram*.

For SCL, TMP and ECG, we found no significant general effect of the sympathetic activity before the sympatric-ANS trigger observation (*movement* and *paralinguistic*). Tables 2, 3 also show the comparison of times T1-T5 vs. T6 (50–60 s)., and thus presents B-ANS that distinguishes event time from time points of the previous minute. Table 2 shows the sympathetic response prior to the observation *movement* and Table 3 prior to the *paralinguistic* observation trigger. These results clearly show that the significant sympathetic reaction can be demonstrated prior to all observation points (*movement* and *paralinguistic*) for the fEMG_C and fEMG_Z.

Supplementary Tables 3, 4 show the comparison of times T2-T6 vs. T1 (0–10 s) and thus identify B-ANS that present the earliest significant change in relation to the baseline (T1).

**Figure 3:**
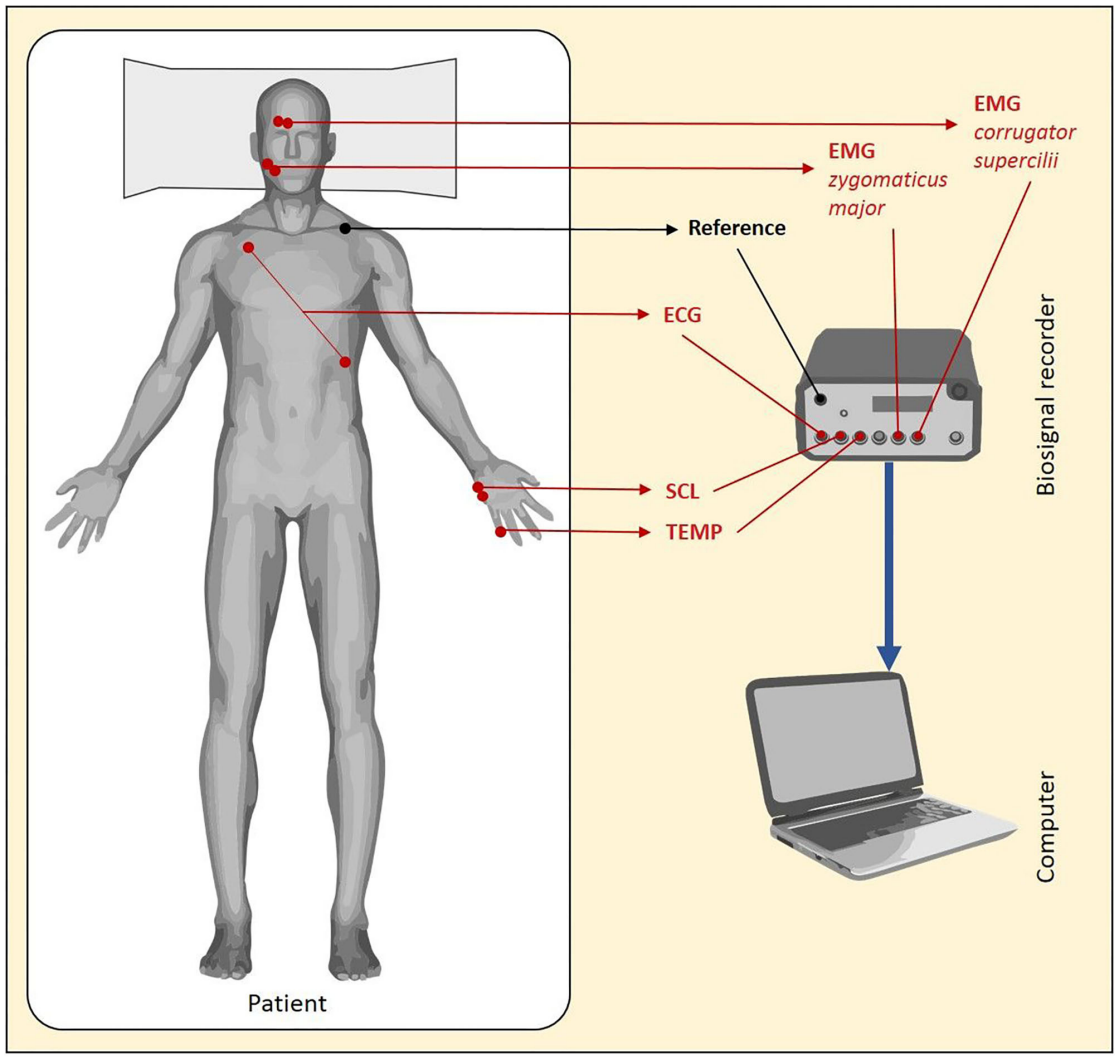
Mean fEMG activity of the facial muscle *corrugator supercilii* over time during the minute before and after the expression of paralinguistic sounds.

Supplementary Table 3 shows that 10 s prior to the observation *movement*, and Supplementary Table 4 shows that 30 s prior to the observation of *paralinguistic* sounds, fEMG_C and fEMG_Z differ significantly from the baseline. This finding is visualized in Figure 3.

### Secondary Endpoints

The aim of the secondary endpoints was to identify other important factors that negatively influence the examiner's satisfaction with the sedation. Interestingly, there was no significant correlation between the mean sedation score (MOAAS), which was measured every 3 min, and the CSSI (*r* = −0.018, *p* = 0.85). All procedures were performed with the use of propofol. Only during 4 (3.6%) procedures was midazolam additionally administered. There was a significant correlation between the propofol dose (mean 6.6 mg ± 5.3 mg per min) relative to procedure time and the CSSI (*r* = −0.190, *p* = 0.045). The total propofol dose (mean 210 mg ± 168) showed just a trend toward significance regarding the correlation to the CSSI (*r* = −0.184, *p* = 0.052). Neither the total number of sedation applications (*r* = −0.181. *p* = 0.056, mean applications per procedure 9.8 ± 3.9), the relative number of sedation applications per procedure time (*r* = −0.152, *p* = 0.073 nor mean applications per min 0.3 ± 0.1) showed a significant correlation to CSSI. The duration of the procedure and especially the time the examiner spent in order to reach the cecum might influence the satisfaction with the procedure. Indeed, the cecal intubation time (*r* = −0.229; *p* = 0.023; mean time to reach the cecum 9.69 ± 5.15 min) rather than the total duration of the procedure (*r* = −0.048; *p* = 0.612; mean procedure time 22.54 ± 16.73 min) significantly correlated with the CSSI. The polypectomy represents a critical event during a colonoscopy due to the high level of concentration and the orchestrated interaction required between nurse and physician to remove the polyp in one piece and manage complications like bleeding. In total, polyps were removed from 37 patients. The mean number of polyps removed was 2.3 ± 1.7. A subgroup analysis involving only the 37 polyp removal cases revealed that there is a correlation between a higher number of polyps removed and the CSSI (*r* = −0.346, *p* = 0.036). Due to the different challenges associated with a polypectomy, we further analyzed the polyp resection time. The annotated time was available for 26 procedures. Concordant to the number of polyps removed, the duration did significantly correlate to the CSSI (*r* = 0.517, *p* = 0.007; mean duration of polyp removal 150 s ± 406). In order to assess the influence the nurse performing the sedation has on the satisfaction of the examiner, we analyzed the working experience of the nurse and the individual employee. There were 9 nurses who participated in the study. Their endoscopy experience ranged from 1 to 20 years. There was no correlation between the work experience of the nurse and the CSSI (*r* = 0.02, *p* = 0.833). Interestingly, our results showed a significant correlation between individual nurses and the CSSI (*p* = 0.009). Due to the finding that the cecum intubation time had an influence on the CSSI and that nurses do not only administer the sedation but also assist with the abdominal compression during the procedure, we further analyzed their work experience and the cecal intubation time. Neither the years of working experience of the nurse (*r* = 0.027, *p* = 0.790) nor the experience of the examining physician (*r* = 0.077, *p* = 0.453, range of endoscopy experience 4 to 19 years) correlated with the cecal intubation time.

## Discussion

NAPS using single propofol bolus applications is the standard procedure for performing colonoscopies in many countries worldwide. Although it is a safe method and widely accepted by patients and examiners, there is room for improvement regarding the timing of the propofol bolus application (30). In this study we aimed to identify observer-reported pain events (movements and paralinguistic sounds) and correlate these events with the satisfaction of the examiner as measured by the CSSI. We were able to show that although the patients were satisfied with the procedure in the vast majority of cases (mean PSSI 93.7 ± 10.9), a substantial proportion of the examiners expressed a lower satisfaction (mean CSSI 82.5 ± 15.4) and rated 26 procedures (23.2%) with a value of 70 or lower. In comparison, only 3.4% of the patients rated the procedure with a PSSI of lower than 70. This is consistent with other studies that compared the perceptions of those two groups (16, 31). Additionally, there was no significant correlation between the two scores. Lin et al. (16) compared CAPS using fentanyl and propofol with a non-CAPS approach using a midazolam-fentanyl sedation. The mean PSSI of 93.7 and the CSSI of 83.8 in the non-CAPS procedures were similar to our values. Pambianco et al. (31) compared CAPS using fentanyl and propofol with a non-CAPS sedation method based on benzodiazepine and an opioid. The mean values of PSSI 87.7 and 92.3 respectively, were comparable with our results as well. Interestingly, the mean CSSI of 76.3 in the non-CAPS group was lower than our values. One main reason might be the different drugs used for sedation in this study. Propofol induced less pain during and after the procedure than benzodiazepine (15) which seemed to beneficially influence patient movement during the procedure (32).

Other factors that were analyzed and that might influence CSSI were various time measurements of the procedure. The mean duration of the procedure of 22.54 min was similar to previous studies ranging from 19.5 to 25.0 min (9, 31, 32) and interestingly did not influence the CSSI. In contrast, the mean time of 9.69 min that was required to reach the cecum of mean 9.69 min influenced the CSSI and was similar to the published values of 8.0 to 9.33 min (33, 34). Thus, a longer time to reach the cecum seems to play a more important role with regard to satisfaction than the total length of the procedure.

The mean propofol dose used was 210 mg, which is in the range of previously published studies ranging from 180 to 347 mg (9, 12, 13, 32) as well. There was only a trend toward significance between the total propofol dosage and the CSSI.

Interestingly, there was no correlation between the working experience of the nurse with the CSSI but instead there was a significant correlation between the individual nurse and the CSSI. The importance of individuals is in line with a publication that identified individual endoscopists as modifiable factors associated with pain during and after colonoscopy (15).

Our main aim was to correlate observer-reported pain events, which include movements and paralinguistic sounds, to CSSI. The explicit observer-reported movements, to our knowledge, were only assessed by Schroeder et al. (32) and showed that the use of propofol instead of midazolam with fentanyl resulted in a lower rate of movement during the procedure. An accompanying effect was that the examiner rated the colonoscopy for which a propofol sedation was chosen as less difficult. Thus, pain during the endoscopy seems to negatively influence the examiner's satisfaction with the procedure.

With the existing automated CAPS method according to (16) it has already been shown that physiological masses of capnometry are suitable to automatically detect the sedation depth (35). In the present study, an attempt was made to operationalize the sympathetic/parasympathetic (distress level) regulation via B-ANS during a colonoscopy by means of external observation points (trigger: movement and paralinguistics). It was shown that a significant fEMG activity of the facial expression was activated 20–30 s prior to the observation trigger of movement and paralinguistic sounds.

The limitations of the current study include the fact that there is no gold standard for detecting pain during an endoscopy. We tried to overcome this limitation by using an observer-reported pain recognition system that was performed only by one investigator during the entire study and that made it possible to log every event in a custom-made application on a PC tablet. Overall, B-ANS (36–38) and the EMG, in particular, are so-called surrogate parameters which are susceptible to artifacts caused by pulling on the electrode cables or by touching the electrodes, e.g., when the patient moves or is repositioned. Furthermore, electromagnetic fields can also influence the quality of the signals. Especially with regard to the EMG, the signal quality depends on the expertise with which the sensors are applied. The fact that, with regard to the SCL and the temperature, the results only go in the expected direction of the effect could be related to the influence of propofol. Due to the window size of 10 s, only limited heart rate variability features can be calculated. A feature such as the RMSSD (measure of vagus-mediated heart rate variability) only makes sense for a 60 s window or longer.

An evaluation of multiple factors that might have influenced the satisfaction of the examiner in addition to observer-reported pain events was specified by the protocol and performed in this study. Some of those factors are the duration of the procedure, the time it took to reach the cecum or the years of professional experience of the assisting nurse. Still, other factors not accessed such as the size and location of the polyps that were resected might have additionally influenced the satisfaction of the examiner.

Additional limitations include the heterogeneous study population with various indications for the procedure. Only patients with ASA I and II and thus a relatively healthy patient cohort were included. Conventional statistics to detect changes in the baseline that predict the occurrence of an event were used. Artificial intelligence (AI) algorithms based on deep learning might additionally detect patterns that can identify such changes. In order to actually train and test artificial intelligence algorithms via B-ANS, more recording times are necessary, which means that a large sample of about ≥500 patients would be required.

## Conclusion

In summary, the current study shows that there are multiple factors that influence the satisfaction of examiners with the sedation during a colonoscopy as measured by the CSSI. Movements and paralinguistic sounds are two factors that correlate with a lower satisfaction with the sedation. Additionally, movements and paralinguistic sounds were identified as two factors that can be adequately measured and their occurrence possibly predicted by EMG of facial muscles. Thus, this implies that an early identification or prediction of movements and paralinguistic sounds by facial EMG can be used to potentially prevent their occurrence by applying sedation drugs. Further work, including a prospective evaluation of the administration of propofol once a defined fEMG threshold has been reached, might shed more light on the use of B-NAS in NAPS. Furthermore, a larger amount of multimodal B-ANS need to be collected in order to apply, train and test AI algorithms.

It would be conceivable to use the highly sensitive facial expression activity to record the so-called activity thresholds of the fEMG (measured in μV), which are at approx. > 6 μV with regard to facial muscle tone (baseline corresponds to ~3 μV). From a visionary point of view, this could—for routine clinical practice—be integrated into a system for monitoring distress activity: e.g., when the fEMG threshold is exceeded, the system indicates with a light or sound (“pain alarm”) that Propofol needs to be injected.

However, it would also be conceivable to expand the CAPS system such that the fEMG facial expression is combined with capnometry signals, thus representing a hybrid technology.

## Data Availability Statement

The datasets generated for this article are not readily available due to German data protection regulations. Requests to access the datasets should be directed to steffen.walter@uni-ulm.de.

## Ethics Statement

The studies involving human participants were reviewed and approved by Ethical committee of Ulm, Germany (No 278/18). The patients/participants provided their written informed consent to participate in this study.

## Author Contributions

AH: study concept and design, interpretation of results, statistical analysis, drafting of the manuscript, acquisition of data, data collection, and critical revision. SGr: interpretation of results, statistical analysis, drafting of the manuscript, and critical revision. SGo: acquisition of data, data collection, and critical revision. NM, SF, and BW: support of study materials and software, and critical revision. SW: study concept and design, interpretation of results, statistical analysis, drafting of the manuscript, support of study materials and software, critical revision. All authors contributed to the article and approved the submitted version.

## Conflict of Interest

The authors declare that the research was conducted in the absence of any commercial or financial relationships that could be construed as a potential conflict of interest.

## Abbreviations

ANS: Autonomic Nervous System
ASA: American Society of Anesthesiologists
B-ANS: Biosignals of the Autonomic Nervous System
CAPS: Computer Assisted Propofol Sedation
CRC: Colorectal Cancer
CSSI: Clinician Satisfaction with Sedation Instrument
fEMG: facial Electromyography
fEMG_C: facial Electromyography of musculus corrugator supercilii
fEMG_Z: facial Electromyography of musculus zygomaticus major
ECG: Electrocardiogram
MOAAS: Modified Observer Assessment of Alertness and Sedation
NAPS: Nurse Assisted Propofol Sedation
PSSI: Patients Satisfaction with Sedation Instrument
SCL: Skin Conductance Level
TMP: Temperature.
