# Supplementary material for: Autonomous Nervous Response During Sedation in Colonoscopy and the Relationship With Clinician Satisfaction
Source: Front Med (Lausanne). 2021 Jun 16;8:643158. doi: 10.3389/fmed.2021.643158 (PMC8242168; doi:10.3389/fmed.2021.643158)
Supplement: Supplementary file 1 [file Data_Sheet_1.docx]

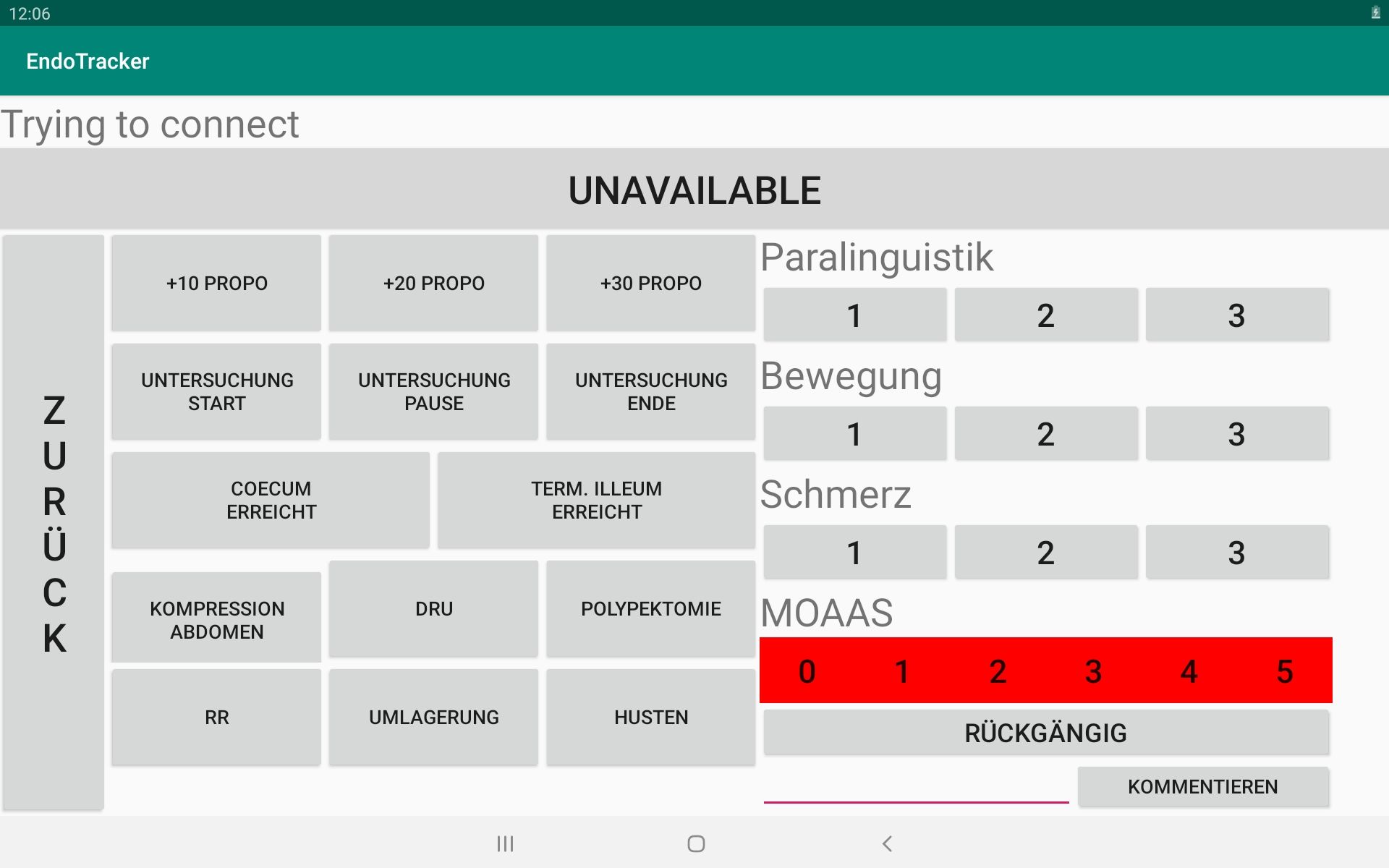


**Supplementary figure 1**: Screen shot of the custom-made application running on the tablet PC device and enabling to track observer reported pain events during the endoscopic procedure.


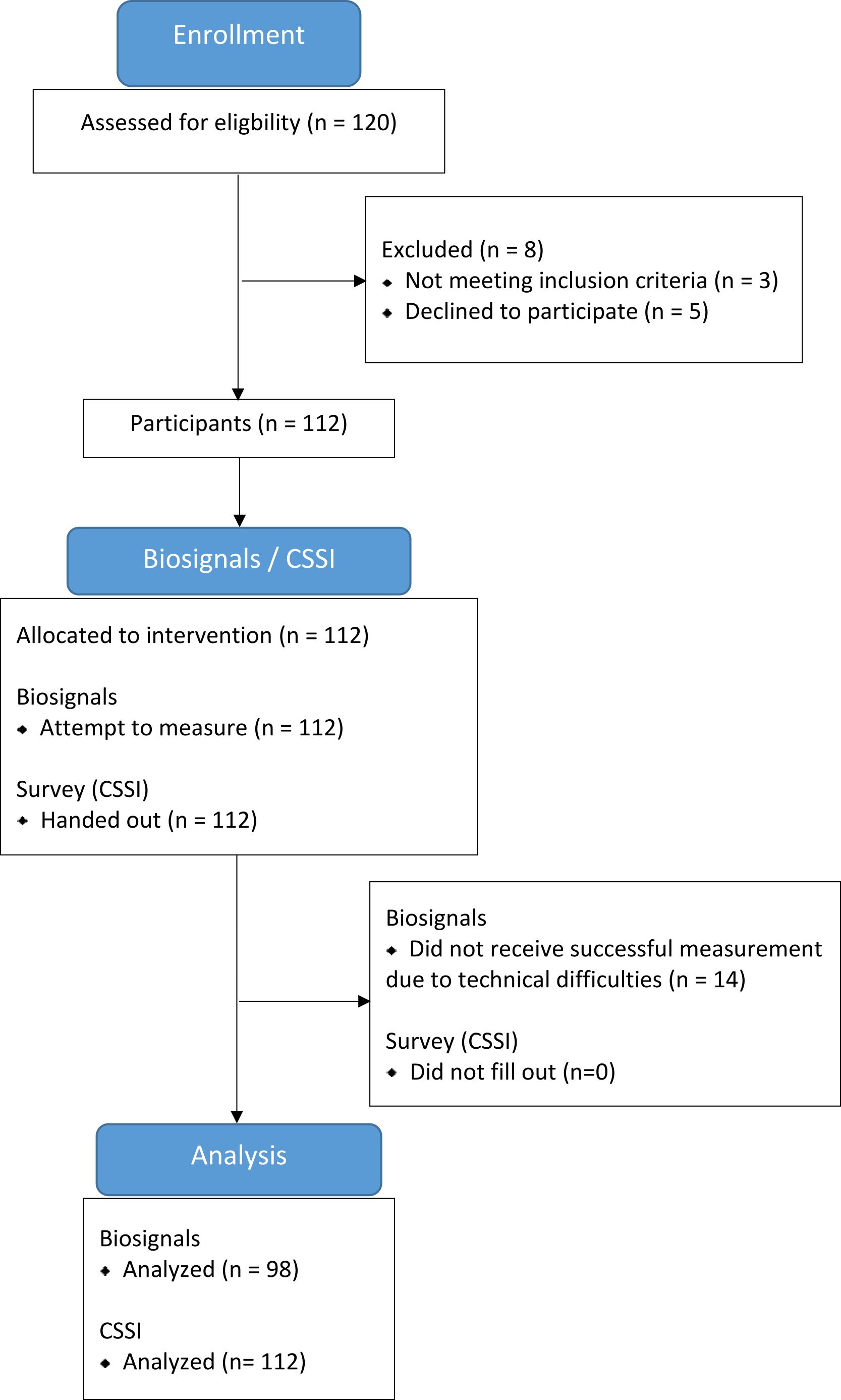


**Supplementary figure 2**: Patient flow chart

| **Indication for endoscopy** |  |
| --- | --- |
| colorectal cancer screening and polyp removal, n(%) | 32 (29) |
| inflammatory bowel disease, n(%) | 30 (27) |
| abdominal pain, n(%) | 17 (15) |
| suspected gastrointestinal bleeding or anemia, n(%) | 13 (12) |
| diarrhea, n(%) | 9 (8) |
| Other, n(%) | 24 (21) |

**Supplementary table 1:** Indication for endoscopy.

| **Sedation characteristic** |  |
| --- | --- |
| Examinations with sedation, n (%) | 112 (100) |
| Sedation with propofol, n (%) | 112 (100) |
| Sedation with propofol only, n (%) | 109 (97.3) |
| Sedation with propofol combined with midazolam, n (%) | 3 (2.7) |
| Total propofol dose, mean +/- SD, mg | 210 +/- 168 |

**Supplementary table 2:** Drugs used for sedation during colonoscopy. SD = standard deviation

|  | fEMG_C (N = 93) | | | fEMG_Z (N = 92) | | | SCL (N = 82) | | | TMP (N = 94) | | | ECG (N = 85) | | |
| --- | --- | --- | --- | --- | --- | --- | --- | --- | --- | --- | --- | --- | --- | --- | --- |
| before observation | mean | wilcoxon | p-value | mean | wilcoxon | p-value | mean | wilcoxon | p-value | mean | wilcoxon | p-value | mean | wilcoxon | p-value |
| T1 (00-10s) | .678 |  |  | .431 |  |  | -.103 |  |  | 28.735 |  |  | 428.507 |  |  |
| T2 (10-20s) | .524 | T1-T2 | .000 | .361 | T1-T2 | .007 | -.099 | T1-T2 | .340 | 28.746 | T1-T2 | .776 | 430.639 | T1-T2 | .426 |
| T3 (20-30s) | .651 | T1-T3 | .021 | .453 | T1-T3 | .304 | -.102 | T1-T3 | .636 | 28.759 | T1-T3 | .565 | 432.292 | T1-T3 | .076 |
| T4 (30-40s) | .653 | T1-T4 | .158 | .445 | T1-T4 | .321 | -.104 | T1-T4 | .441 | 28.779 | T1-T4 | .463 | 433.808 | T1-T4 | .039 |
| T5 (40-50s) | .843 | T1-T5 | .079 | .541 | T1-T5 | .043 | -.098 | T1-T5 | .776 | 28.811 | T1-T5 | .166 | 433.584 | T1-T5 | .015 |
| T6 (50-60s) | 1.421 | T1-T6 | .000 | .724 | T1-T6 | .000 | -.050 | T1-T6 | .175 | 28.819 | T1-T6 | .290 | 428.678 | T1-T6 | .631 |

**Supplementary table 3**: Autonomous sympathetic activity before movement observation (n = 94) to six events T1 vs. T2-6. fEMG_C ≙ Electromyography-Corrugator; fEMG_Z ≙ Electromyography-Zygomaticus; SCL ≙ Skin Conductance Level; TMP ≙ Temperature; ECG ≙ Electrocardiogram

**Supplementary table 4**: Autonomous sympathetic activity before paralinguistic observation to six events T1 vs. T2-6. EMG_C ≙ Electromyography-Corrugator; EMG_Z ≙ Electromyography-Zygomaticus; SCL ≙ Skin Conductance Level; TMP ≙ Temperature; ECG ≙ Electrocardiogram

|  | fEMG_C (N = 59) | | | fEMG_Z (N = 58) | | | SCL (N = 51) | | | TMP (N = 59) | | | ECG (N = 55) | | |
| --- | --- | --- | --- | --- | --- | --- | --- | --- | --- | --- | --- | --- | --- | --- | --- |
| before observation | mean | wilcoxon | p-value | mean | wilcoxon | mean | wilcoxon | p-value | mean | wilcoxon | mean | wilcoxon | p-value | mean | wilcoxon |
| T1 (00-10s) | .8143 |  |  | .5131 |  |  | -.0608 |  |  | 27.9784 |  |  | 439.5736 |  |  |
| T2 (10-20s) | .8448 | T1-T2 | .424 | .5522 | T1-T2 | .287 | -.0199 | T1-T2 | .729 | 27.9903 | T1-T2 | .922 | 445.1325 | T1-T2 | .323 |
| T3 (20-30s) | 1.0500 | T1-T3 | .298 | .5846 | T1-T3 | .819 | -.0219 | T1-T3 | .606 | 28.0106 | T1-T3 | .640 | 444.6785 | T1-T3 | .325 |
| T4 (30-40s) | 1.1222 | T1-T4 | .026 | .6515 | T1-T4 | .060 | -.0259 | T1-T4 | .736 | 28.0348 | T1-T4 | .551 | 445.9821 | T1-T4 | .272 |
| T5 (40-50s) | 1.3029 | T1-T5 | .006 | .6419 | T1-T5 | .004 | -.0106 | T1-T5 | .261 | 28.0531 | T1-T5 | .645 | 447.7451 | T1-T5 | .124 |
| T6 (50-60s) | 1.7350 | T1-T6 | .000 | .8775 | T1-T6 | .000 | .0576 | T1-T6 | .085 | 28.0807 | T1-T6 | .757 | 448.0119 | T1-T6 | .932 |
